# Why Pimping Works: The Neurophysiology of Emotional Memories

**DOI:** 10.7759/cureus.64237

**Published:** 2024-07-10

**Authors:** Med Jimson D Jimenez, Pranish Kantak, Jeffrey Raskin

**Affiliations:** 1 Medicine, Chicago Medical School, Rosalind Franklin University of Medicine and Science, North Chicago, USA; 2 Neurological Surgery, Henry Ford Health System, Detroit, USA; 3 Neurological Surgery, Northwestern University Feinberg School of Medicine, Chicago, USA; 4 Pediatric Neurological Surgery, Ann & Robert H. Lurie Children’s Hospital of Chicago, Chicago, USA

**Keywords:** neurophysiology, emotional memory, learning, hippocampus, pimping

## Abstract

A time-honored medical ritual that combines emotion and cognition into a seamless consolidation of lucid memories is a feared teaching method in medical education. The resulting neurophysiology is explained from a neurosurgeon’s perspective - equal parts guilt and dread as a prescription for an improved and sustained trainee fund of knowledge. Much of the available literature published with regard to pimping explores its pedagogy and use in medical practice. This review aims to explore the neurobehavioral and biological aspects of pimping in why it remains a popular teaching model. We describe the neuromodulatory process of integrating emotions and memory as observed during pimping. Additionally, we explore the neuronal pathways and circuits involved in memory encoding, consolidation, and retrieval. Finally, we explored the effects of this methodology as it is currently used in the United States medical education system.

## Introduction and background

Uncovering unique methods of teaching students is one of the essential roles of a mentor during the arduous path to becoming a practicing physician. The hierarchical realm of American medical academia has a history of training generations of physicians with unique traditions that outsiders might find puzzling at first glance. Abraham Flexner describes it upon a 1916 visit to Johns Hopkins, “Rounded with Osler today. Riddles house officers with questions. Like a Gatling gun. Welch says medical students call it ‘pimping.’ Delightful” [1].

The term “pimping” refers to the interaction between a student and their instructor, typically a resident or an attending physician where they pose a rapid-fire series of questions to the student with the intent to teach. It is an interaction that was popularized in modern medical history as a form of medical art [1]. This act of public interrogation provides a learning paradigm where the most long-lasting and potent memories are encoded. For example, I remember every gruesome second that I fidgeted under the gaze of my neurosurgeon mentor in medical school, unable to recall the innervation of the platysma. I felt embarrassed. The manifestation of emotion provides a contextual backdrop that contributes significantly to the lasting consolidation of the memory. We hope that, through this narrative, students and educators will learn to appreciate the unique methodology behind some of the most stress-inducing experiences in medical education. Herein, we will describe potential neural pathways for the formation of lucid memories when professors, residents, and medical students participate in the time-honored, yet sometimes horrifying tradition, of “pimping.”

## Review

Basic concepts in memory

**Figure 1:**
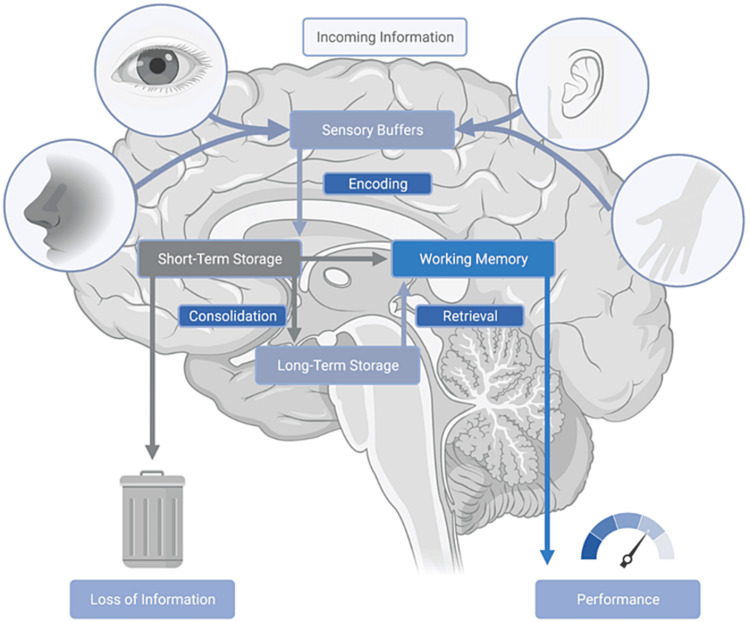
Sensory modalities underlying memory formation.

For pimping to be a successful teaching tool, it must be able to modulate memory encoding, consolidation, and retrieval. Figure 1 schematically depicts the interplay between these modalities. Memory encoding is the critical first step to creating a new memory. It allows for incoming information, in any perceptual form, to be converted into a neural construct that is stored as short-term memory [2]. Consolidation refers to the transfer of short-term information to long-term storage. As will be discussed below, it is during consolidation that long-term potentiation (LTP) plays an integral role. Retrieval is defined as the recovery of memories from long-term storage. Pimping, through its interplay with the limbic and sympathetic nervous systems, affects each of these modalities [3].

Neuroscience: a field of serendipitous discoveries

Great advances in the neurophysiology of memory are credited to the 1953 case of HM, a 27-year-old man who suffered intractable seizures treated with bilateral medial temporal lobectomies and resection of bilateral hippocampi. Immediately following surgery, HM was unable to form new memories, although he was able to remember memories that were already encoded. He retained these deficits for the entirety of his life. In studying his case, the hippocampal formation was identified as the neural machine that consolidates declarative “explicit” memory - the same type of memory augmented by pimping [4]. While the hippocampus is generally considered the memory “hub” in the brain, many different neural structures serve to orchestrate memory formation. We will discuss the hippocampus and other members of the limbic system, as these are considered to be the most critical in emotional memory formation [5].

Synaptic plasticity and LTP

In the 1940s, psychologist Donald Hebb laid the groundwork for what is now colloquially known as Hebbian theory. This dogma states that repeated, simultaneous activation of presynaptic and postsynaptic neurons increases the synaptic strength between them [6]. This principle is the underlying hypothesis defining “synaptic plasticity.” Synaptic plasticity was further characterized in the hippocampus physiologically as LTP.

Synaptic plasticity occurs when glutamate released from presynaptic neurons activates the N-methyl-D-aspartate (NMDA) type glutamate receptors in the postsynaptic neuron [7,8]. Activation of the NMDA through the binding of glutamate along with a voltage-dependent removal of magnesium will allow calcium influx in the post-synaptic terminal [9,10]. The calcium influx along with a CAMKII-dependent cascade will result in the insertion of α-amino-3-hydroxy-5-methyl-4-isoxazolepropionic acid (AMPA) receptors in the postsynaptic membrane [11-13]. Comparing both NMDA and AMPA glutamate activation, NMDA is slower and is responsible for modulating the synaptic response while AMPA is faster and is responsible for most of the rapid excitatory synaptic transmission [14]. The resulting increased density of AMPA receptors in the post-synaptic membrane highlights the Hebbian theory of synaptic plasticity emphasized in the hippocampus. This newly strengthened neuron pathway will be more likely to generate a faster and stronger action potential response from the subsequent glutamate release highlighting synaptic strengthening and the resulting plasticity. LTP within the hippocampal formation is believed to be the neurophysiological correlate to declarative memory consolidation [15].

Amygdala and emotional tagging

In addition to the hippocampus, we must consider the amygdala when discussing the acquisition and retention of emotionally encoded memories. This concept deemed “emotional tagging” revolves around amygdala activation during the encoding of emotionally arousing events, such as during a particularly embarrassing public interrogation of facial muscle innervation. The activation tags this memory as essential and facilitates increased synaptic plasticity in other areas of the brain, namely the hippocampus [5].

Emotional tagging and fear conditioning is a form of operant conditioning best recognized by the experiments of B. F. Skinner with the delivery of food to rats. He found that rats placed in a box can be trained to increase behaviors such as pressing a lever for a reward such as food and to decrease certain behaviors to avoid punishment such as being shocked [16,17]. In the case of pimping, students learn to change their behavior because of a reward like being praised or getting a letter of recommendation from their overseer as a form of positive reinforcement or for the avoidance of being embarrassed as a form of negative reinforcement. Once the emotionally arousing event of pimping has been established, the medical student avoids the outcome of being embarrassed by emotionally tagging the correct answers to memory. The next time that the student is pimped, the student may avoid the feeling of embarrassment by correctly answering the question. Naturally, this will condition the student to come prepared with the material and can be reinforced over time. Fear conditioning is rapidly acquired and has a stable and long-lasting behavioral change as was shown in the student example. The conditioning allows the student to retain the answer to the questions through the concept of “emotional tagging.” This is the “Law of Effect” forming the basis of operant conditioning where responses that produce satisfying effects are more likely to occur again while discomforting effects are less likely to occur [18].

Emotional regulation of the sympathetic nervous system

Systemically released chemical mediators of the sympathetic nervous system cause vasodilation, facial flushing, sweating, tachycardia - and something else. Stimulated by the unknown, students experience an emotion: embarrassment. This feeling is associated with a state of emotional arousal that facilitates heightened attention. The Papez circuit also referred to as the medial limbic circuit, is involved with the production of emotional expression. This circuit starts and ends at the hippocampal formation (subiculum) and encompasses many neural structures including the mammillothalamic tract, anterior thalamic nucleus, and entorhinal cortex. It is known that damage to structures within the medial limbic circuit can result in memory and language impairment [19]. Additionally, sympathetic regulation by way of β-adrenergic receptors plays an important role in mood regulation and memory [20]. Adrenergic receptors in the limbic system mediate sympathetic neuromodulation through a most interesting brainstem structure: the locus coeruleus (LC) [21].

An integral part of the emotional memory machinery

Latin for “blue spot,” the LC in the dorsal pons acts as the most significant producer of norepinephrine (NE) in the brain. The LC innervates nearly every neural structure, thereby affecting wide-ranging cognitive and emotional functions. It is connected to the dorsal hippocampus and basolateral amygdala by strong projection fibers [22]. In embarrassment, the LC directs extreme attention to the task at hand by attenuating background activity and accentuating the task-specific signal within the prefrontal cortex, hippocampi, and amygdala complexes. Upon activation, NE projections from the LC to the hippocampus are activated, and NE is released into the hippocampus, facilitating LTP between neurons. Simultaneously, NE projections from the LC activate the amygdala and promote the induction of LTP by amygdala projection fibers to the hippocampus [5,23,24]. Finally, NE can also serve to coordinate population-level neuronal responses through hippocampal oscillatory patterns, namely: sharp-wave ripples (SWRs) [25].

LC also drives oscillatory patterns in the hippocampus: SWRs

**Figure 2:**
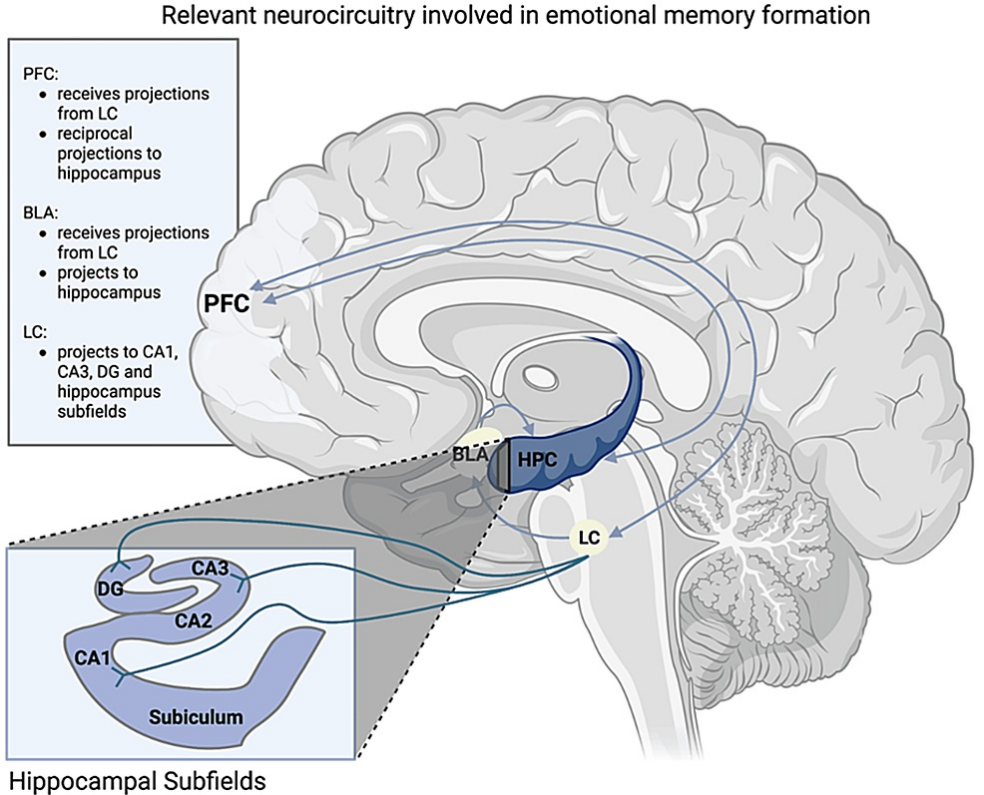
Neurophysiological correlates of emotional memory formation. The locus coeruleus (LC) modulates structures involved in emotional memory formation via long-term potentiation and set-point modulation. PFC = prefrontal cortex; BLA = basolateral amygdala; HPC = hippocampus; CA = cornu ammonis; DG = dentate gyrus.

SWRs consist of waves of neuronal excitation that originate in the CA3 subfield of the hippocampus and may spread to the CA1 subfield. These oscillatory patterns are predominant during slow-wave sleep and immobility. During SWRs, there is evidence that similarly suggests neurons fire when behaviors are being learned. In layman's terms, during SWRs, the newly encoded information is being “replayed,” which may further serve to potentiate memory consolidation [26,27]. Interestingly, in vitro experiments in rodents suggest that the introduction of β-adrenergic receptor agonists in the hippocampus facilitates the induction of SWRs and LTP. These experiments further support the role of NE release from LC projections in the hippocampus’s ability to consolidate memory [25]. Figure 2 shows the reciprocal nature and complex relationship between the neural circuitry discussed in this paper.

Post-traumatic stress disorder (PTSD) and pimping

Pimping can lead to long-term consolidation of memory through emotional tagging. PTSD is explored as there is an abundance of literature surrounding its neurophysiology and its marked similarity to pimping. By finding similarities between the two models, there will be more insight and understanding of the physiology of pimping. As far as we know, there is no functional imaging explicitly studying pimping while there are vast data available studying PTSD. PTSD is marked by heightened emotional and arousal responses, along with difficulty in extinguishing learned fear similar to what is seen during the pimping process [28]. Functional magnetic resonance imaging of patients with PTSD shows that there are shared core networks involving the amygdala, and prefrontal cortex in learning, processing, and extinction of fear conditioning in human studies [29,30]. Additionally, it is known that activation of the amygdala in patients with PTSD is associated with fear and hyperarousal [31]. Positron emission tomography (PET) studies have also shown that subjects with heightened activity in the amygdala remember more of the emotional component compared to subjects with less activity [32]. It has also been shown that retrieval of traumatic memories by individuals with PTSD activates limbic areas such as the amygdala and temporal cortex [33,34]. These imaging studies highlight the importance of an emotional component with memory and help understand the basis of pimping and why this pedagogy remains popular in medical institutions. The vast literature that is available on PTSD can be utilized to pave the path to understanding the physiology and neurobehavioral aspects of pimping.

Review of literature: pimping and medical education

A PubMed search through January 2023 was conducted using the search terms ((medical education) AND (learn*) AND (pimp*)). The aim of this search was meant to understand the role of pimping within the medical education system. Papers were included only if they were published in the English language. Papers must be cases, clinical reports, reviews or meta-analyses. Articles that are purely commentary in nature or supplementary were excluded from the manuscript. Articles that did not talk about the impact of pimping on residents, students, or on the United States medical education system were excluded. This generated initially 22 articles through PubMed. After screening the generated articles, we have included three articles in our analysis.

Pimping in the medical practice

One study from the University of California Irvine looked at the effects of pimping when utilized on teaching rounds by faculty on medical students [35]. They found through the use of a survey that students described this experience as stressful even under benign teaching environments and affected their learning and performance. Both students and faculty found that a malignant environment such as those when pimping was done in the presence of patients detrimentally affected medical student learning [35]. Another study from the University of Michigan found through a survey of 140 students and 112 faculty that medical students (89%) and faculty (97%) agreed that pimping was valuable in medical education [36]. The majority of students were found to prefer being asked more questions than none at all with researchers arguing for the use of the Socratic method in medical education [36]. Another study from Johns Hopkins University School of Medicine looked into how much of their faculty utilized pimping techniques in their medical education practices [37]. They created a numeric pimping score which classified those in the upper quartile of the score as faculty that utilized pimping in medical education. Researchers found that younger, male participants, specialists were significantly more likely to be utilizing pimping practices. They found through survey techniques that almost half of their faculty who responded were also helped in their own learning through pimping [37].

## Conclusions

The full elucidation of memory stabilization and storage is still an ongoing investigation that will hopefully share insights into the pathogenesis of memory disorders such as dementia once fully understood. It begs a question knowing that the hippocampus is not the final site for long-term declarative memory utilized in pimping of how this might come about in the neocortex and how the eventual independence from the hippocampus and limbic system comes about. Studies in mice models suggest that spatial memory during non-REM sleep, as characterized using an electroencephalogram (EEG), allows stored memory in the hippocampus to be ultimately projected to higher-order functional structures in the neocortex.

Additionally, the framework of PTSD as a model to understand pimping is interesting and much underexplored in our literature review. Parallels between activation pathways and the role of the amygdala in emotional processing beg the question of the long-term effects of pimping. Many medical institutions in the United States utilize pimping in their medical education daily for their students. From our short systematic review of the utilization of pimping in the United States, there is some evidence of positive benefits of pimping to both the learner and educator. It is interesting to note that the literature predominantly discusses the benefits of incorporating this methodology and teaching style; this may shed light on why such practices that date back many centuries are still used in practice today. Further studies into how frequent and the degree of pimping would be helpful to explore any negative effects and implications on the health and well-being of the learner. There may be a link between burnout and physician mental health with the use of pimping that remains much undiscovered as evidenced by the lack of literature available in this area.

The modulation of the LC by emotion and sympathetic arousal and its subsequent control over the limbic system promotes the Hebbian state of synaptic plasticity. LTP of specific synapses causes the consolidation of a long-lasting and robust memory. I am certain I will know this for the rest of my life: the facial nerve innervates the platysma. Embarrassment is a complex emotion, incorporating a sense of guilt, fear, and motivation, paired with a heightened sense of arousal. It is within this framework that the benefit of “pimping” lies. We should use this emotion and teaching style, not because of its inherent complexity or our desire to be malignant towards our students, but because it defines the context in which arousal and focused attention can serve to modulate declarative memory formation.
